# Morphometrics predicts the differential regurgitant fraction in bilateral pulmonary arteries of patients with repaired tetralogy of fallot

**DOI:** 10.1007/s10554-023-03035-1

**Published:** 2024-02-16

**Authors:** Hui-Chung Teng, Yi-Chun Chen, Yung-Lin Chen, Ken-Pen Weng, Jun-Yen Pan, Ming-Hua Chang, Hsiu-Wen Cheng, Ming-Ting Wu

**Affiliations:** 1 Department of Radiology, Kaohsiung Veterans General Hospital, No. 386, Dazhong 1st Rd., Zuoying District, Kaohsiung, 813414 Taiwan; 2 Department of Nursing, Mei Ho University, Pingtung, Taiwan; 3 School of Medicine, College of Medicine, National Yang Ming Chiao Tung University, Taipei, Taiwan; 4 Congenital Structural Heart Disease Center, Department of Pediatrics, Kaohsiung Veterans General Hospital, Kaohsiung, Taiwan; 5 Division of Cardiovascular Surgery, Department of Surgery, Kaohsiung Veterans General Hospital, Kaohsiung, Taiwan; 6 Institute of Clinical Medicine, National Yang Ming Chiao Tung University, Taipei, Taiwan

**Keywords:** Differential regurgitant fraction, Pulmonary regurgitation, Tetralogy of fallot, Cardiovascular magnetic resonance, Pulmonary artery angle

## Abstract

In patients with repaired tetralogy of Fallot (rTOF), the regurgitant fraction (RF) in left pulmonary artery (LPA) and right pulmonary artery (RPA) is usually unequal. The morphometrics may play a crucial role in this RF discrepancy. Cardiovascular MR of 79 rTOF patients and 20 healthy controls were retrospectively enrolled. Forty-four from the 79 patients were matched in age, sex and body surface area to the 20 controls and were investigated for: (1) phase-contrast flow of main pulmonary artery (MPA), LPA, and RPA; (2) vascular angles: the angles between the thoracic anterior–posterior line (TAPL) with MPA (θ_M–AP_), MPA with RPA (θ_M–R_), and MPA with LPA (θ_M–L_); (3) cardiac angle, the angle between TAPL and the interventricular septum; (4) area ratio of bilateral lung and hemithorax regions. Compared with the 20 controls, the 44 rTOF patients exhibited wider θ_M–AP_, sharper θ_M–L_ angle, and a smaller θ_M–L_/θ_M–R_ ratio. In the 79 rTOF patients, LPA showed lower forward, backward, and net flow, and greater RF as compared with RPA. Multivariate analysis showed that the RF of LPA was negatively associated with the θ_M–L_/θ_M–R_ ratio and the age at surgery (R^2^ = 0.255). Conversely, the RF of RPA was negatively associated with the left lung/left hemithorax area ratio and cross-sectional area (CSA) of LPA, and positively associated with CSA of RPA and MPA (R^2^ = 0.366). In rTOF patients, the RF of LPA is more severe than that of RPA, which may be related to the vascular morphometrics. Different morphometric parameters are independently associated with the RF of LPA or RPA, which may offer potential insights for surgical strategies.

## Introduction

Pulmonary regurgitation (PR) is the most important complication in patients with repaired tetralogy of Fallot (rTOF). Although variable degrees of PR related to rTOF can be tolerated for many years, PR in some rTOF patients becomes severe and is associated with dilatation and dysfunction of the right ventricle (RV), and subsequently the left ventricle, therefore contributing to long-term morbidities, including exercise intolerance, atrial and ventricular arrhythmia, congestive heart failure, and possibly, sudden death [1, 2].

Cardiovascular MR (CMR) is regarded as part of standard care in serial follow-up studies of rTOF patients [3]. Phase-contrast magnetic resonance imaging (PC-MRI) is accepted as the imaging modality of choice for the quantification of PR due to its accurate and reproducible measurement of the flow volume, velocity, and blood flow patterns [4], even in patients with suspected peripheral pulmonary stenosis [5]. By using the contrast-enhanced magnetic resonance angiography (CE-MRA) with 3-dimensional (3D) reconstruction, the morphometrics of the pulmonary arteries and cardiopulmonary anatomy can be well assessed and analyzed.

Previous studies have shown that the branch pulmonary arteries in rTOF patients often have differential RF and contribute unequally to total PR [6–8]. The RF of LPA is usually larger than that of RPA. The cause of differential RF is still unclear. Kato et al. [9] discovered that an enlarged and levorotated heart is related to compression of the left lung, which in turn contributes to increased diastolic flow reversal in LPA. Chern et al. [10] found that in rTOF patients, the regurgitation initially occurs in LPA because of the small angle between LPA and MPA, and a recirculation zone impeding the forward pulmonary blood flow is observed within LPA during the acceleration of systole. However, no large-scale studies have been reported to comprehensively evaluate the contributory factors of differential branch RF in rTOF patients.

We hypothesized that the vascular morphometrics may play an important role in the mechanism of differential RF in bilateral pulmonary arteries of rTOF patients. In this retrospective CMR study, we compared the morphometrics between rTOF patients and the normal controls. We anticipated that the results may provide morphophysiological insights that may be useful for surgical consideration in the management of TOF or rTOF patients.

## Materials and methods

### Patients

Between January 2004 and October 2018, patients who underwent CMR surveillance after total repair of tetralogy of Fallot at Kaohsiung Veterans General Hospital were retrospectively reviewed. There were 118 cases identified within the Research Information System. Among them, thirteen cases exhibited incomplete CMR image sequences or suboptimal image quality for evaluation. A total of 26 patients were excluded due to the following causes: rTOF with pulmonary valve replacement (n = 4), post-endovascular stent placement in pulmonary arteries (n = 2), significant branch pulmonary artery stenosis (defined as > 50% reduction in diameter compared to the proximal abutting vessel [11], n = 8) and incomplete surgical procedure records (n = 12). As a result, 79 rTOF patients were enrolled in this study. Patient characteristics were shown as Table 1.

**Table 1:** Characteristics of the study population

Characteristics	n = 79
Age	15.9 ± 9.6
Male: female	50: 29
*Tetralogy of Fallot*	
with pulmonary stenosis, n (%)	62 (78%)
with pulmonary atresia, n (%)	17 (22%)
Palliation before total repair (mBTS), n (%)	19 (24%)
Transannular patch, n (%)	47 (59%)
Age at total repair, years	1.7 [3.1, 0.2–29.1]*
Age at CMR study, years	17.5 [15.2, 1.2 ~ 46.1]*
Interval between surgery and CMR, years	16.2 [13.5, 0.5–28]*

*median [interquartile range, range]

*CMR* cardiovascular magnetic resonance, *mBTS* modified Blalock-Taussig shunt

We also reviewed our previous CMR case collections of healthy volunteers for use as a normal control group. The inclusion criteria for this group were: 1. Age and sex similar to that of the patient group; 2. Absence of symptoms and signs or established diagnosis of cardiopulmonary diseases during the initial scan and subsequently reconfirmed. As a result, a normal control group of 20 healthy individuals was retrospectively enrolled in this study. However, there was a difference of age, sex and body surface area (BSA) between the two groups. To further remove these potential confounding factors, we then selected 44 from the 79 rTOF patients with matched age, sex and BSA to the 20 controls for comparison. As a result, there was no statistically significant difference of age, sex and BSA between the two groups.

The Research Ethics Board of our institute approved this study. All patients or patients' parents, depending on the patients’ age, provided written informed consents.

### MRI protocol

CMR studies were performed on a 1.5-T system (Signa CV/I, GE Healthcare, Milwaukee, Wisconsin). PC-MRI acquisition was applied for MPA, RPA, and LPA. The effective temporal resolution was regulated to allow for 20 reconstructed phases per cardiac cycle. Besides, CSA of the pulmonary arteries were generated from the selected contours encircling MPA, RPA, and LPA on the magnitude images of the phase contrast acquisition. Acquired PC-MRI data were processed in an independent workstation. The flow analysis software (CVI42, Circle Cardiovascular Imaging INC., Calgary, Canada) was used to generate flow volume-time curves of the MPA, RPA, and LPA. The flow measurement included forward flow volume (FFV), backward flow volume (BFV), net flow volume (NFV), and RF. NFV was defined as FFV minus BFV. RF was defined as BFV divided by FFV.

### Morphometric definitions

**Fig. 1:**
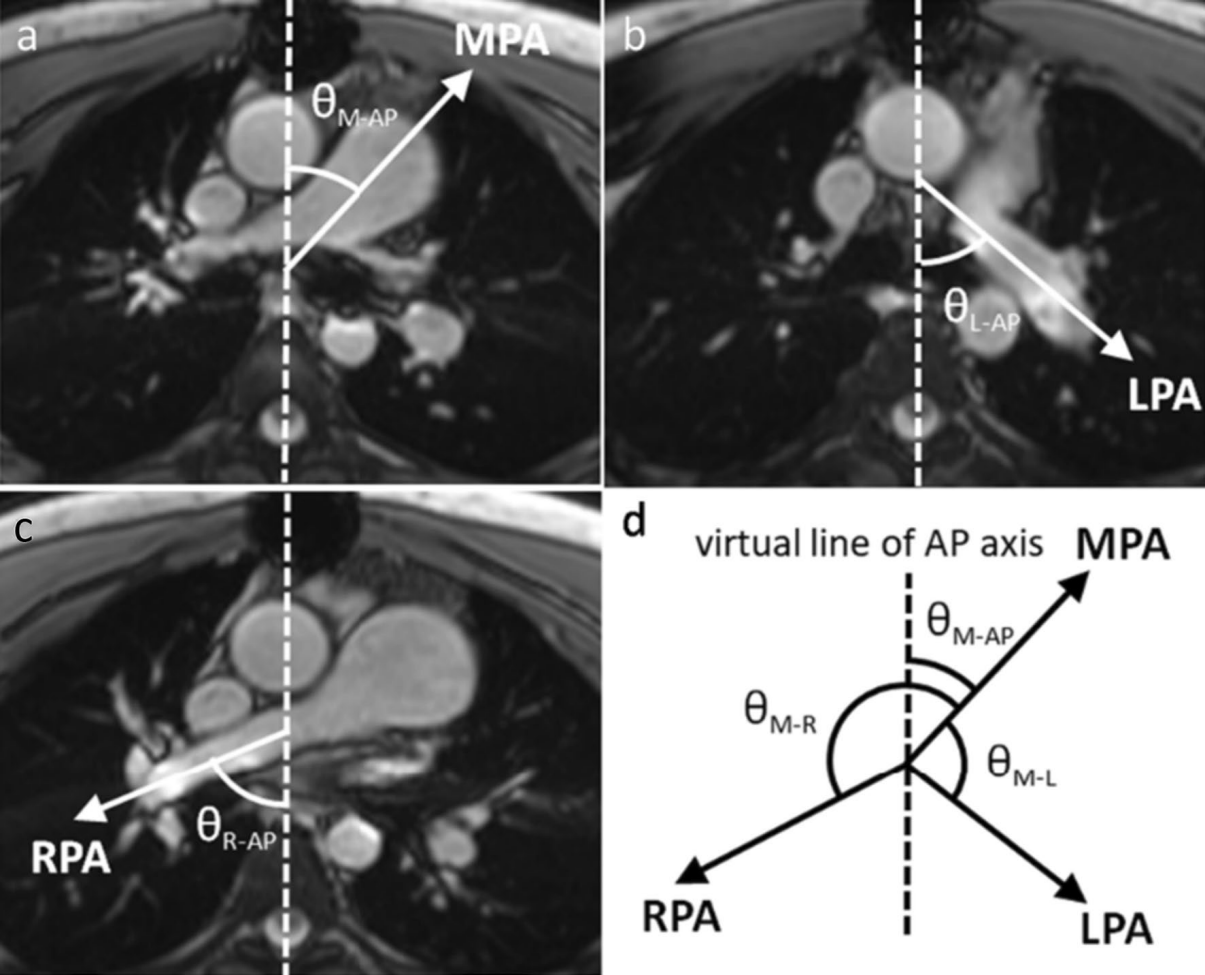
Axial plane images for measuring pulmonary artery angles **a** The angle between the thoracic anterior–posterior line and main pulmonary artery was measured, defined as θ_M–AP._ **b** The angle between the thoracic anterior–posterior line and left pulmonary artery was measured, defined as θ_L–AP_. **c** The angle between the thoracic anterior–posterior line and right pulmonary artery was measured, defined as θ_R–AP. _**d** The angle between main pulmonary artery and right pulmonary artery (defined as θ_M–R_) can be calculated as follows: θ_M–R_ = 180—θ_R–AP_ + θ_M–AP._ The angle between main pulmonary artery and left pulmonary artery (defined as θ_M–L_) can be calculated as follows: θ_M–L_ = 180—θ_M–AP_—θ_L–AP_ *AP* anterior–posterior, *LPA* left pulmonary artery, *MPA* main pulmonary artery, *RPA* right pulmonary artery

Source images of 3D CE-MRA images were reconstructed on the workstation (AW server 3.2 Ext. 4.0, GE Healthcare, Milwaukee, Wisconsin). Images were reconstructed in three standardized projections as shown in Fig. 1 for the measurement of pulmonary artery angles. We measured the angle between the thoracic anterior–posterior line and MPA (defined as θ_M–AP_, Fig. 1a), between the thoracic anterior–posterior line and LPA (defined as θ_L–AP_, Fig. 1b), and between the thoracic anterior–posterior line and RPA (defined as θ_R–AP_, Fig. 1c) on axial images. MPA, RPA, and LPA were identified individually because they may not lie in the same axial plane. The angle between the MPA and RPA (defined as θ_M–R_, Fig. 1d) can be calculated as follows: θ_M–R_ = 180—θ_R–AP_ + θ_M–AP._ The angle between the MPA and LPA (defined asθ_M–L_, Fig. 1d) can be calculated as follows: θ_M–L_ = 180—θ_M–AP_–θ_L–AP._

**Fig. 2:**
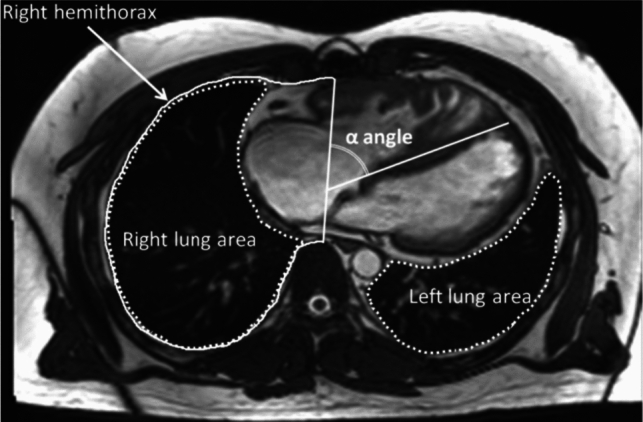
Axial plane image for measuring the cardiac angle (α angle) and calculating the lung area ratio

According to the report of Kato et al. [9] (Fig. 2), we measured the cardiac angle (referred to as α angle) and calculated the lung area ratio. Measurements were taken on the axial plane image displaying the largest cardiac surface area. The cardiac angle was defined as the angle between the thoracic anterior–posterior line and the interventricular septum. Lung area ratio was calculated as the quotient of lung area to the ipsilateral hemithorax area ratio.

### Statistical analysis

The parameters were expressed as median (interquartile range) or mean ± standard deviation according to normality tests and analyzed by paired samples t-test. Correlation between parameters was evaluated by Pearson correlation analysis. Because the RF of LPA, RPA, and MPA were normally distributed, multivariate linear regression was used for predicting the effect of morphological and clinical profiles on the RF. All *p* < 0.05, 2-sided, were considered statistically significant. All analyses were done with IBM SPSS Statistics, version 22.0.

## Results

### PC-MRI flow measurement

The flow volumes of the LPA are significantly lower than those of RPA in terms of FFV, BFV, and NFV, while the CSA are similar between LPA and RPA. In contrary, the RF of LPA was 0.42, which was higher than the RF of RPA (0.30) (*p* < 0.001). The details are listed in Table 2.

**Table 2:** Blood flow characteristics and cross-sectional area in pulmonary arteries of rTOF patients (n = 79)

	MPA		LPA		RPA		paired-t test of LPA and RPA
MRI flow measurement	median	IQR	median	IQR	median	IQR	*p* value
Forward flow volume, ml/min	5124	2809	1359	1310	2690	1944	** < 0.001**
Backward flow volume, ml/min	1872	2690	522	728	717	1228	**0.012**
Net flow volume, ml/min	3010	1644	822	871	1905	1143	** < 0.001**
Regurgitant fraction	0.4	0.26	0.42	0.31	0.3	0.28	** < 0.001**
Cross-sectional area, mm^2^	614	430	157	153	186	141	0.684

*p* values < 0.05 were defined as statistically significant

*IQR* interquartile range, *LPA* left pulmonary artery, *MPA* main pulmonary artery, *RPA* right pulmonary artery, *rTOF* repaired tetralogy of Fallot

### Pulmonary artery angle

For the matched 44 rTOF patients to the normal control group, θ_M–AP_ and θ_M–R_ were larger in matched rTOF patients than in the normal control group (*p* < 0.001), whereas θ_M–L_ was smaller (*p* < 0.001). As a result, the θ_M–L_/θ_M–R_ ratio was significantly smaller in the matched rTOF patients group than in the normal control group (*p* < 0.001) (Table 3).

**Table 3:** Comparison of pulmonary artery angles between selected rTOF patients and the normal control group

	rTOF patients	normal control	*p* value
	n = 44	n = 20	
age	22.5 ± 4.2	21.3 ± 0.7	0.074
sex (M/F)	27/17	10/10	0.394
BSA (m^2^)	1.7 ± 0.2	1.6 ± 0.2	0.261
FFV of MPA, ml/min	5919 (2768)	4525.20 (1084)	**0.004**
BFV of MPA, ml/min	2666 (3027)	54 (62)	** < 0.001**
NFV of MPA, ml/min	3586 (1610)	4445 (1064)	** < 0.001**
RF of MPA	0.44 (0.28)	0.01 (0.01)	** < 0.001**
FFV of LPA, ml/min	1763 (1081)	2118 (831)	0.072
BFV of LPA, ml/min	669 (959)	18 (54)	** < 0.001**
NFV of LPA, ml/min	1125 (941)	2054 (848)	** < 0.001**
RF of LPA	0.44 (0.40)	0.01 (0.02)	** < 0.001**
FFV of RPA, ml/min	29,975 (1662)	2185 (800)	**0.016**
BFV of RPA, ml/min	974 (1431)	5 (19)	** < 0.001**
NFV of RPA, ml/min	1959 (794)	2185 (769)	0.064
RF of RPA	0.33 (0.34)	0.002 (0.01)	** < 0.001**
CSA of MPA, mm^2^	748 (364)	492 (166)	**0.001**
CSA of LPA, mm^2^	193 (184)	197 (90)	0.908
CSA of RPA, mm^2^	223 (93)	177 (67)	**0.036**
θ_M–AP_, degree	34.1 ± 13.4	12.5 ± 6.9	** < 0.001**
θ_M–L_, degree	108.1 ± 22.2	129.0 ± 10.3	** < 0.001**
θ_M–R_, degree	145.3 ± 13.8	125.4 ± 8.4	** < 0.001**
θ_M–L_/θ_M–R_ ratio	0.76 ± 0.21	1.03 ± 0.11	** < 0.001**

The parameters were expressed as median (interquartile range) or mean ± standard deviation according to normality tests; *p* values < 0.05 were defined as statistically significant

*BFV* backward flow volume, *BSA* body surface area, *CSA* cross-sectional area, *FFV* forward flow volume, *LPA* left pulmonary artery, *MPA* main pulmonary artery, *NFV* net flow volume, *RF* regurgitant fraction, *RPA* right pulmonary artery, *rTOF* repaired tetralogy of Fallot, *θ*_*M–AP*_ the angle between the thoracic anterior–posterior line and main pulmonary artery, *θ*_*M–L*_ the angle between main pulmonary artery and left pulmonary artery, *θ*_*M–R*_ the angle between main pulmonary artery and right pulmonary artery

### The relationship between multiple variables and regurgitant fraction

**Fig. 3:**
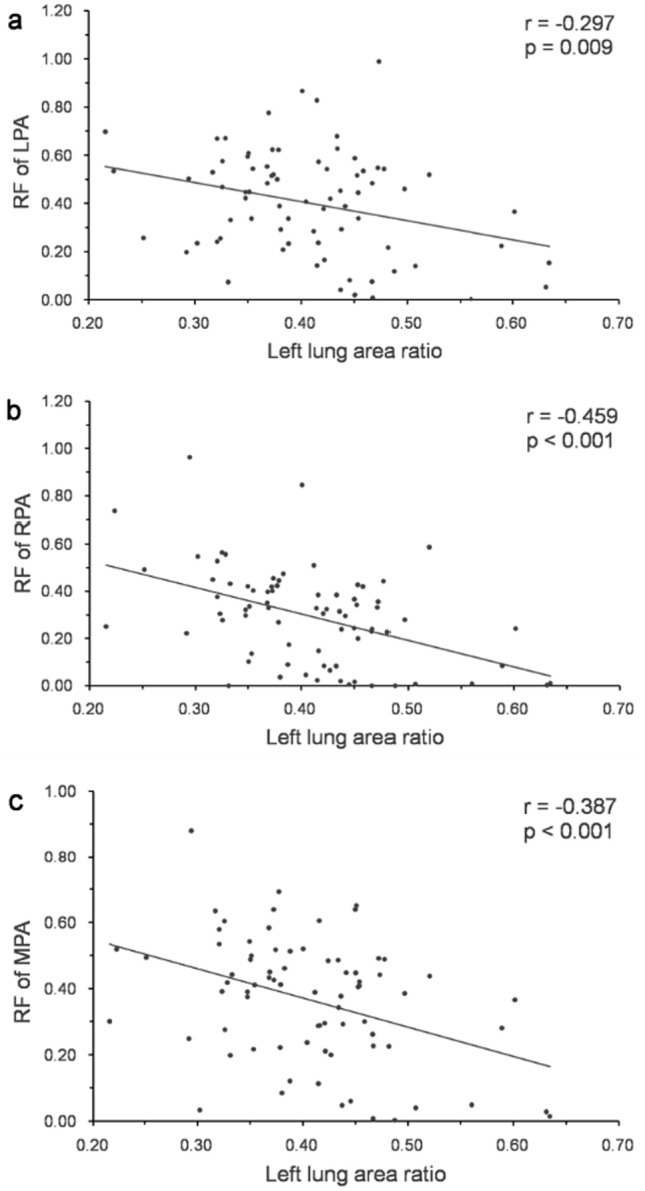
Correlation between regurgitant fraction and left lung area ratio. *LPA* left pulmonary artery, *MPA* main pulmonary artery, *RF* regurgitant fraction, *RPA* right pulmonary artery

**Fig. 4:**
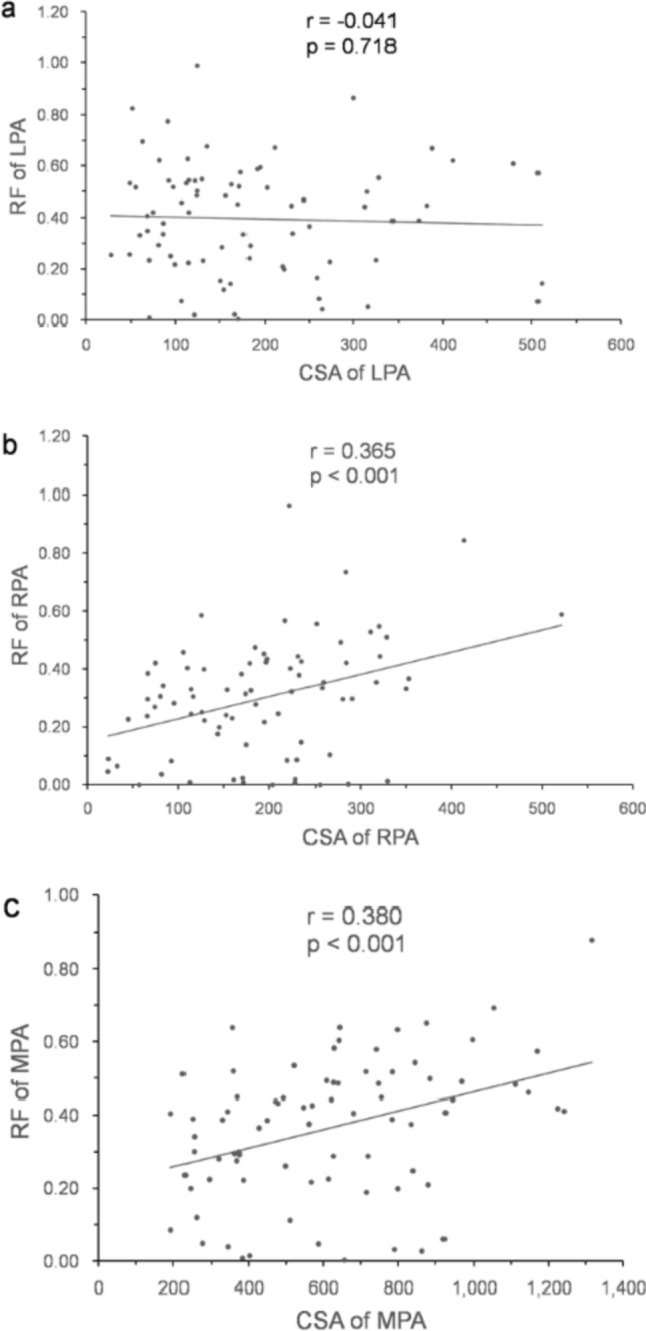
Correlation between regurgitant fraction and cross-sectional area. *CSA* cross-sectional area, *LPA* left pulmonary artery, *MPA* main pulmonary artery, *RF* regurgitant fraction, *RPA* right pulmonary artery

**Fig. 5:**
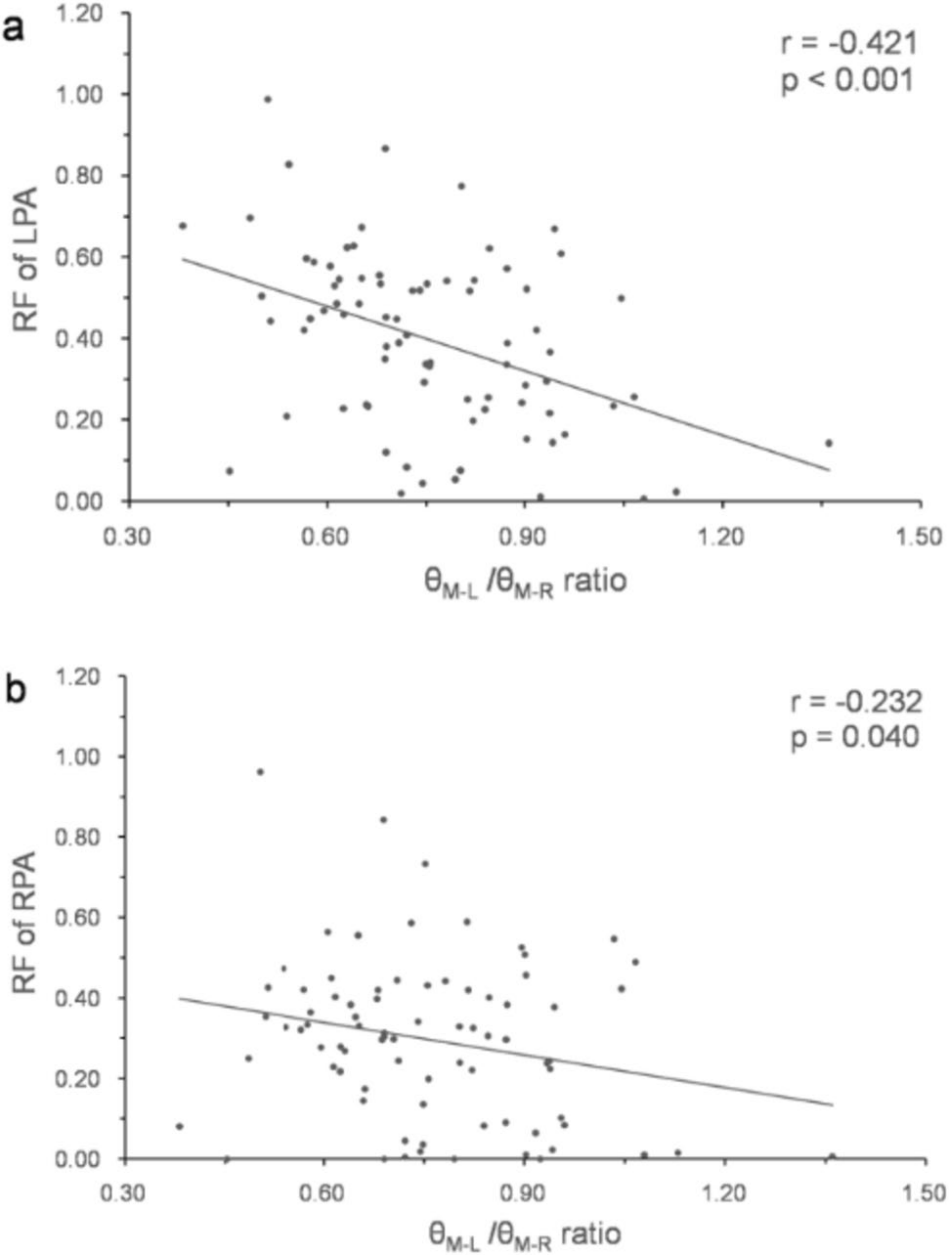
Correlation between regurgitant fraction and θ_M–L_/θ_M–R_ ratio. *LPA* left pulmonary artery, *RF* regurgitant fraction, *RPA* right pulmonary artery, *θ*_*M–L*_ the angle between main pulmonary artery and left pulmonary artery, *θ*_*M–R*_ the angle between main pulmonary artery and right pulmonary artery

The relationships between time interval factors, flow factors, morphometric factors, and RF are shown in Table 4. The RF of LPA, the RF of RPA, and the RF of MPA correlated with each other significantly. Left lung area ratio correlated inversely with the RF of LPA (Fig. 3a) and α angle, the same result as the report of Kato et al. [9]. Left lung ratio also correlated inversely with the RF of RPA (Fig. 3b) and the RF of MPA (Fig. 3c). The RF of LPA did not correlate with CSA of LPA (Fig. 4a), whereas the RF of RPA correlated with CSA of RPA (Fig. 4b), and the RF of MPA correlated with CSA of MPA (Fig. 4c). The RF of LPA correlated inversely with θ_M–L_/θ_M–R_ ratio (Fig. 5a) and age at surgery, and the RF of RPA also correlated inversely with θ_M–L_/θ_M–R_ ratio (Fig. 5b).

**Table 4:** The relationships between time interval factors, flow factors, morphogen metric factors, and RF

	Univariate analysis (n = 79)						Multivariate analysis (n = 79)									
	RF of LPA		RF of RPA		RF of MPA		RF of LPA		RF of LPA		RF of RPA		RF of RPA		RF of MPA	
							model 1^a^		model 2^b^		model 1a^a^		model 2^b^		model 2^b^	
	r^c^	*p* value	r^c^	*p* value	r^c^	*p* value	β^d^	*p* value	β^d^	*p* value	β^d^	*p* value	β^d^	*p* value	β^d^	*p* value
Time interval factors																
birth-CMR	0.148	0.194	−0.032	0.776	0.045	0.691										
OP-CMR	−0.052	0.646	0.057	0.618	0.136	0.231										
birth-OP	−0.277	0.013	−0.212	0.061	−0.179	0.114	−0.211	0.013	−0.297	0.004						
Flow factors																
FFV of LPA																
BFV of LPA																
NFV of LPA																
RF of LPA			0.535	< 0.001	0.537	< 0.001										
FFV ofRPA																
BFV of RPA																
NFV of RPA																
RF of RPA	0.535	< 0.001			0.707	< 0.001										
FFV of MPA	0.379	< 0.001	0.424	< 0.001												
BFV of MPA	0.526	< 0.001	0.666	< 0.001			0.638	< 0.001								
NFV of MPA	−0.003	0.979	−0.095	0.403												
RF of MPA	0.537	< 0.001	0.707	< 0.001							0.580	< 0.001				
morphogeo metric factors																
CSA of LPA	−0.041	0.718	−0.123	0.282							−0.289	< 0.001	−0.322	0.002		
CSA of RPA	0.038	0.739	0.365	< 0.001							0.283	0.001	0.310	0.006		
CSA of MPA	0.157	0.166	0.364	< 0.001	0.380	< 0.001	−0.265	0.013					0.230	0.042	0.313	0.004
Left lung area ratio	−0.297	0.009	−0.459	< 0.001	−0.387	< 0.001					−0.218	0.007	−0.393	< 0.001	−0.322	0.003
Right lung area ratio	0.065	0.578	0.246	0.032	0.138	0.236										
α angle	0.235	0.041	0.393	< 0.001	0.322	0.005										
θ_M -A P_	0.372	< 0.001	0.333	0.003	0.347	0.002										
θ_M -L_	−0.354	0.001	−0.175	0.124												
θ_M -R_	0.327	0.003	0.194	0.086												
θ_M -L_/θ_M-R_ ratio	−0.421	< 0.001	−0.232	0.040			−0.359	< 0.001	−0.434	< 0.001						
adjusted R^2^							R ^2^ = 0.511		R ^2^ = 0.255		R ^2^ = 0.613		R ^2^ = 0.366		R ^2^ = 0.223	

*p* values < 0.05 were defined as statistically significant

model 1^a^: a factors including the MPA flow parameters; model 2^b^: a factors excluding the MPA flow parameters; r^c^ the correlation coefficient; β^d^ standardized beta coefficient

*BFV* backward flow volume, *birth-CMR* time interval between birth and cardiovascular magnetic resonance performed, *birth-OP* time interval between birth and surgery, *CSA* cross-sectional area, *FFV* forward flow volume, *LPA* left pulmonary artery, *lung area ratio* the quotient of lung area to the ipsilateral hemi thorax area ratio, *MPA* main pulmonary artery, *NFV* net flow volume, *OP-CMR* time interval between surgery and cardiovascular magnetic resonance performed, *RF* regurgitant fraction, *RPA* right pulmonary artery, *α angle* the angle between the thoracic anterior–posterior line and the interventricular septum,θ_M–AP_ the angle between the thoracic anterior–posterior line and main pulmonary artery, θ_M–L_ the angle between main pulmonary artery and left pulmonary artery, θ_M–R_ the angle between main pulmonary artery and right pulmonary artery

The multivariate linear regression analysis involved the creation of two distinct models. Model 1 encompassed all factors, including the MPA flow parameters, whereas model 2 excluded the MPA flow parameters from the independent variables. For predictors of the RF of LPA, the model 1 (Table 4) included CSA of MPA, θ_M–L_/θ_M–R_ ratio, age at surgery, and BFV of MPA (R^2^ = 0.511); the model 2 included θ_M–L_/θ_M–R_ ratio and age at surgery (R^2^ = 0.255). For predictors of the RF of RPA, the model 1 included the RF of MPA, left lung area ratio, CSA of LPA, and CSA of RPA (R^2^ = 0.613); the model 2 included left lung area ratio, CSA of LPA, CSA of RPA, and CSA of MPA (R^2^ = 0.366). In addition, the predictors of the RF of MPA include left lung area ratio and CSA of MPA (R^2^ = 0.223).

## Discussion

In this study, we investigated the morphology of pulmonary arteries and their relationship with RF in branch pulmonary arteries of rTOF patients by CMR. The main findings of our study can be summarized as follows: (1) The morphometrics of rTOF patients is characteristically different from that of the normal control group: (1a) The course of MPA in rTOF patients tilted more to the left side. (1b) rTOF patients had larger θ_M–R_, smaller θ_M–L_and smaller θ_M–L_/θ_M–R_ ratio. (2) The RF of RPA and the RF of LPA could be predicted with and without known MPA flow parameters in rTOF patients. (2a) With MPA flow parameters, both the RF of RPA and the RF of LPA can be predicted by MPA flow parameters and morphometrics. (2b) Without known MPA flow parameters, both can still be predicted by morphometrics alone with a less accuracy.

The underlying mechanism of complicative dynamic interactions of pulmonary arteries, ventricular function and lung condition is still not underscored. The proposed one by McElhinney et al. [12] sugested that (1). PR after repair operation caused the dilatation and elongation of the pulmonary trunk, (2). The right ventricular outflow tract would be transferred superiorly and rotated to the left. This causes the kinking of LPA at its origin from MPA, which may be coordinated by tethering of LPA by the ligamentum arteriosum. As a result, θ_M–L_ is smaller in rTOF patients than in the normal population. Besides, θ_M–R_ becomes more obtuse, causing the θ_M–L_/θ_M–R_ ratio smaller in rTOF patients. Kato et al. [9] also indicated that due to the limited anteroposterior mediastinal space, the enlargement of RV will cause the heart bulging into the left hemithorax and compressing the left lung, which implies the change in branch pulmonary artery angle with the kinking of LPA. The CT-based 3D morphometrics study by Luo et al. [13] showed that θ_M–R_ was larger and θ_M–L_ was smaller in rTOF patients as compared with the normal control group.

We found that none of CSA of all three pulmonary arteries correlates with the RF of LPA, while the bifurcation angles are the key factors. Chern et al. have used an in-vitro numerical pulmonary artery model based on computational fluid dynamics to simulate flow variations in pulmonary arteries after repair of TOF [10]. They showed that regurgitation of LPA occurs earlier than RPA and MPA when θ_M–R_ was set significantly more obtuse than θ_M–L_, which implied that the regurgitation of LPA may be more prolonged and prominent than RPA in that situation. Previous studies have established that theoretically there are optimal values for vascular angles and diameters that make the arterial bifurcation more efficient physiologically [15, 16]. The optimal diameters and branching angles of pulmonary arteries are those that produce the minimal value of cost to drive the blood flow [17]. Zhang et al. [18] found that acute LPA angle is related to adverse hemodynamic performance, such as vortices formation, relative high wall shear stress, decreased flow distribution to the left lung and increased energy loss. Kinking or stenosis of the branch pulmonary artery would increase right ventricular afterload, contribute to vortices formation in the center of the vessel lumen, and cause the acceleration of pulmonary regurgitation. Echocardiography study by Zhang et al. [14] also showed that the global longitudinal strain of RV was lower for acute LPA angulation than for round and blunt LPA angulation in rTOF patients. Our findings strongly support the concept that the sharper bifurcation angle of LPA is the key factor of increased RF in LPA of rTOF patients.

In contrary to LPA, the RF of RPA was correlated with CSA of RPA, consistent with our previous study [7]. Harris et al. [19] observed a significantly increased RF in the larger branching pulmonary artery as compared with the smaller one, a finding that aligns with our results concerning the RF of RPA. Kato et al. [9] found that the left lung area ratio correlated inversely with the RF of LPA and α angle. As the heart gradually enlarged, it would rotate into the left chest, compressing the left lung. We postulated that the enlarged heart would influence the RF of LPA, RPA, and even MPA, according to the results of this study (Table 4).

The best time for performing total repair of TOF is still controversial in pediatric cardiovascular surgery. The American Association for Thoracic Surgery2022 Expert Consensus Document by Miller et al. [20] concluded that the best age for total repair of TOF lies between 3 and 6 months for asymptomatic infants. However, Tamesberger et al. [21] found that neonatal total repair is associated with more frequent use of transannular patches and reinterventions, which may cause the subsequent variable degree of PR. Borowski et al. [22] found that rTOF patients undergoing transannular patch aged > 5 years had considerably longer redo-free intervals than their younger counterparts, which implied that the younger patients may suffer from more severe degree of PR. These studies may support our findings that total repair performed at a later age is correlated with a reduced degree of RF in the MPA, LPA, and RPA, as revealed through univariate analysis in this study.

In model 1 (incorporating MPA flow parameters) of this study, the RF of both RPA and LPA can be predicted by MPA flow parameters and morphology. In model 2 (excluding MPA flow parameters), the pulmonary bifurcation angle is the key factor for the RF of LPA, whereas CSA of pulmonary arteries and left lung area ratio serve as key factors for the RF of RPA. Model 1 offers a stronger prediction due to the influence of MPA flow and pulmonary artery morphometrics; however, model 2 provides simply morphometrical information for predicting PR in instances where flow parameters are unavailable, such as when the computed tomography angiography is employed in the rTOF patients.

Based on the findings from current and previous studies [9, 19, 23], we postulated the consequent pathophysiology of PR in rTOF patients as follows: After total repair of TOF, relief of RV outflow tract obstruction may result in PR (mainly after transannular repair of TOF). Chronic volume overload from PR leads to RV dilatation and MPA elongation. RV dilatation combined with subsequent left ventricular dilatation prompts the enlarged heart to rotate into the left chest, then compressing the left lung. This compression increases left pulmonary vascular resistance, further augmenting diastolic flow reversal in the LPA [19], and assumedly in the MPA and RPA as well. Besides, MPA elongation causes LPA kinking, consequently increasing the RV afterload and exacerbating PR. As a result, it is crucial to monitor the morphometric configuration of pulmonary arteries and the RV during long-term follow-up after TOF repair in patients with significant pulmonary regurgitation, even when prior assessments revealed no apparent abnormalities.

During surgical intervention, the influence and importance of the branching pulmonary artery angles should be considered. Luo et al. [13] showed that a larger preoperative pulmonary artery bifurcation angle is a morphometric predictor for early reoperation in rTOF patients. To correct the kinking of the LPA with or without stenosis, it is sometimes necessary to remove redundancy at the kink point and reimplant the LPA at a more favorable vascular angle. In most of the time, it is also essential to shorten the dilated and elongated pulmonary trunk. If the ligamentum arteriosum remains intact, it should be separated, as it would restrict the mobility of the LPA and act as a pivot for the twisting of the dilated MPA [12]. Some surgeons have reported that adopting the pulmonary artery angioplasty technique, which utilizes the anterior wall flap of the MPA [24, 25], can effectively deal with acute angles and/or stenosis of the LPA during total repair of TOF; our findings agree with their suggestions.

The current study has some limitations. Firstly, the case number of this study is medium, which is insufficient to be age-based subgroup analysis. Increasing case number of different ages may reveal different morphometric predictors in different stage of age. Second, although we found an association between the morphometrics and the RF of LPA and RPA, the causal relationship is not clear. Conducting a longitudinal study on the same cases may provide a better interpretation. Third, we used a single axial plane to represent the lung and cardiac ventricular volume instead of 3D volume measurement of the whole lung and heart. However, we aimed to employ accepted method and keep our method simple and referrable. Forth, we used conventional 2D phase-contrast flow measurement. Future study using 4D flow may disclose novel parameters that carry better clinical impact. Fifth, the mechanisms of regurgitant flow may be influenced by other unchecked factors, such as peripheral pulmonary arterial resistance, lung parenchymal pathology, restrictive physiology of RV, etc., which were not investigated in this study.

## Conclusions

In rTOF patients, the RF of LPA is more severe than that of RPA, which may be related to the vascular morphometrics. Our study indicates that a reduced ratio of θ_M–L_/θ_M–R_ highly associated with increase of RF of the LPA, which may offer potential insights for surgical strategies and follow-up management in patients with TOF.

## Data Availability

The datasets generated during and/or analysed during the current study are available from the corresponding author on reasonable request.

## Abbreviations

3D: 3-dimensional
BFV: Backward flow volume
CE-MRA: Contrast-enhanced magnetic resonance angiography
CMR: Cardiovascular magnetic resonance
CSA: Cross-sectional area
FFV: Forward flow volume
LPA: Left pulmonary artery
MPA: Main pulmonary artery
NFV: Net flow volume
PC-MRI: Phase-contrast magnetic resonance imaging
PR: Pulmonary regurgitation
RF: Regurgitant fraction
RPA: Right pulmonary artery
rTOF: Repaired tetralogy of fallot
RV: Right ventricle
α angle: The angle between the thoracic anterior–posterior line and the interventricular septum
θ_L__–AP_: The angle between the thoracic anterior–posterior line and left pulmonary artery
θ_M__–AP_: The angle between the thoracic anterior–posterior line and main pulmonary artery
θ_M__–L_: The angle between main pulmonary artery and left pulmonary artery
θ_M__–R_: The angle between main pulmonary artery and right pulmonary artery
θ_R__–AP_: The angle between the thoracic anterior–posterior line and right pulmonary artery
